# Glucocorticoid Receptor and β-Catenin Interact in Prostate Cancer Cells and Their Co-Inhibition Attenuates Tumorsphere Formation, Stemness, and Docetaxel Resistance

**DOI:** 10.3390/ijms24087130

**Published:** 2023-04-12

**Authors:** Shannalee R. Martinez, Catherine C. Elix, Pedro T. Ochoa, Evelyn S. Sanchez-Hernandez, Hossam R. Alkashgari, Greisha L. Ortiz-Hernandez, Lubo Zhang, Carlos A. Casiano

**Affiliations:** 1 Center for Health Disparities and Molecular Medicine, Department of Basic Sciences, School of Medicine, Loma Linda University, Loma Linda, CA 92350, USA; 2 Department of Physiology, School of Medicine, University of Jeddah, Jeddah 21589, Saudi Arabia; 3 Lawrence D. Longo MD Center for Perinatal Biology, Department of Basic Sciences, School of Medicine, Loma Linda University, Loma Linda, CA 92350, USA; 4 Department of Medicine, Rheumatology Division, School of Medicine, Loma Linda University, Loma Linda, CA 92350, USA

**Keywords:** β-catenin, docetaxel, glucocorticoid receptor, prostate cancer, stemness, tumorspheres

## Abstract

Therapy resistance hinders the efficacy of anti-androgen therapies and taxane-based chemotherapy for advanced prostate cancer (PCa). Glucocorticoid receptor (GR) signaling mediates resistance to androgen receptor signaling inhibitors (ARSI) and has also been recently implicated in PCa resistance to docetaxel (DTX), suggesting a role in therapy cross-resistance. Like GR, β-catenin is upregulated in metastatic and therapy-resistant tumors and is a crucial regulator of cancer stemness and ARSI resistance. β-catenin interacts with AR to promote PCa progression. Given the structural and functional similarities between AR and GR, we hypothesized that β-catenin also interacts with GR to influence PCa stemness and chemoresistance. As expected, we observed that treatment with the glucocorticoid dexamethasone promotednuclear accumulation of GR and active β-catenin in PCa cells. Co-immunoprecipitation studies showed that GR and β-catenin interact in DTX-resistant and DTX-sensitive PCa cells. Pharmacological co-inhibition of GR and β-catenin, using the GR modulator CORT-108297 and the selective β-catenin inhibitor MSAB, enhanced cytotoxicity in DTX-resistant PCa cells grown in adherent and spheroid cultures and decreased CD44+/CD24– cell populations in tumorspheres. These results indicate that GR and β-catenin influence cell survival, stemness, and tumorsphere formation in DTX-resistant cells. Their co-inhibition could be a promising therapeutic strategy to overcome PCa therapy cross-resistance.

## 1. Introduction

Prostate cancer (PCa) is the most diagnosed male cancer worldwide, with men of African ancestry disproportionately affected [1,2]. In 2023, it is estimated that there will be approximately 288,300 new PCa cases, and an estimated 34,700 men expected to die from the disease in the United States [1]. Despite initial response to androgen deprivation therapy (ADT), patients with recurrent disease develop incurable metastatic castration-resistant PCa (mCRPC), which is defined as disease progression with castrate levels of testosterone [3,4]. Continued ADT plus second-generation androgen receptor (AR) signaling inhibitors (ARSI), such as abiraterone, apalutamide, or enzalutamide, combined with taxane-based chemotherapy with docetaxel (DTX) or cabazitaxel (CBZ), are currently the standard of care for mCRPC [4,5]. Growing evidence supports the development of therapy cross-resistance in mCRPC, where resistance to ARSI may also be associated with resistance to taxane chemotherapy [6].

Several mechanisms contributing to ARSI resistance in mCRPC have been identified, including reactivation of AR signaling through AR amplification, mutations, co-regulator activity, or expression of splice variants such as AR variant 7 (AR-V7) [7,8,9,10]. Signaling through the glucocorticoid receptor (GR, encoded by the *NR3C1* gene) has also emerged as a major contributor to ARSI resistance [11,12,13,14,15,16,17,18,19]. GR is closely related to AR, as both steroid receptors belong to the nuclear receptor superfamily and share significant structural similarity and transcriptomic overlap [11,12]. Prolonged treatment of mCRPC with ARSI results in GR upregulation and downstream expression of both AR and GR target genes [11,12]. This confers ARSI resistance via GR-mediated re-activation of a subset of AR target genes due to the transcriptomic and cistronic redundancy between these two nuclear receptors. GR expression is inversely related to AR expression, both in cellular models and clinical prostate tissues, with GR expression lower in early-stage PCa or therapy-naïve mCRPC cells or tissues due to the repressive function of AR and EZH2-mediated methylation of the GR enhancer [12,19,20]. This suggests that GR signaling is dispensable for prostate function and tumorigenesis until AR is inhibited. The induction of GR during ARSI treatment appears to be mediated by the loss of TLE3, a transcriptional co-repressor that regulates AR-mediated repression of GR [21], as well as the activation of the PI3K/AKT signaling pathway [15]. GR signaling has also been linked to DTX resistance in PCa, possibly by activating anti-apoptotic Bcl-2 family members and Mono Amine Oxidase-A [16,22]. In addition, GR silencing in diverse PCa cell lines reduces the capacity for cancer stem cell (CSC)-like tumorsphere formation [14], consistent with evidence that PCa stemness contributes to DTX resistance [23,24,25,26]. Further, glucocorticoids induce the GR-dependent expression of DTX resistance-associated proteins such as lens epithelium-derived growth factor protein of 75 kD (LEDGF/p75) and Clusterin in a panel of PCa cell lines [27]. These studies provide compelling evidence for the role of GR in both ARSI resistance and taxane chemoresistance, implicating this nuclear receptor in PCa therapy cross-resistance.

Although the molecular mechanisms by which GR promotes cancer stemness and therapy resistance are still under investigation, several hints can be found in its structural and functional similarities to AR. Both receptors physically interact and share similar domain structures, functional ligand-dependent interactomes, and transcriptomic profiles [11,12,20,28,29]. β-catenin, a component of the canonical Wnt signal transduction pathway, is also an interacting partner of AR implicated in resistance to ARSI therapy and taxane chemotherapy in PCa [10,30,31,32,33,34,35,36,37,38]. Through this interaction, β-catenin, acting as a transcription-coactivator, enhances AR-dependent transcriptional activity [30,39]. Activating mutations in the gene encoding β-catenin, *CTNNB1*, have been associated with ARSI resistance in patients with mCRPC [40,41]. β-catenin participates in Wnt signaling during PCa progression and development of therapy resistance [35], and its expression in prostatic tissues mirrors GR expression [14,22,34,42,43,44,45]. Notably, β-catenin transcriptionally activates stemness and chemoresistance-associated genes, including *CD44*, *NANOG*, *MYC*, and *CXCR4* [46,47,48,49,50]. A recent study indicated that inhibition of the Wnt/β-catenin pathway in the presence of DTX in PCa cells downregulated genes linked to chemoresistance [38]. Taken together, these studies also implicate β-catenin in PCa therapy cross-resistance.

Considering the structural and functional similarities between GR and AR [11,12,51], we hypothesized that GR and β-catenin might also interact in PCa cells and together contribute to stemness and DTX resistance. In the present study, we describe this novel interaction and show that pharmacological co-inhibition of these proteins increases sensitivity to DTX while decreasing stemness and tumorsphere formation in mCRPC cells.

## 2. Results

### 2.1. Altered GR and β-Catenin Expression in DTX-Resistant PCa Cells

**Figure 1:**
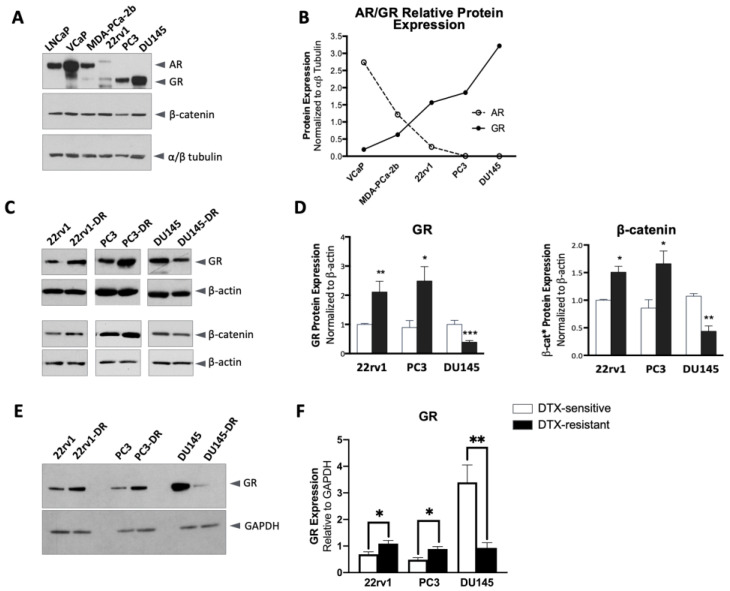
GR and β-catenin are differentially expressed in DTX-resistant PCa cells. (**A**) Total AR and GR expression, detected with specific, non-cross-reactive antibodies in immunoblots, are inversely related, while total β-catenin expression is relatively constant across a diverse panel of PCa cell lines grown under standard cell culture media supplemented with FBS. (**B**) Quantification of total AR and GR expression in selected cell lines shown in panel A. (**C**) Altered total GR and β-catenin protein expression detected by immunoblotting in parental, DTX-sensitive PCa cell lines (22rv1, PC3, and DU145) and their sublines selected for resistance to DTX (DR). The DR cell lines were generated by continuous exposure to incrementally increasing concentrations of DTX (0.1 nM to 10 nM) until stable (>95%) viability was achieved at 10 nM DTX, a pharmacologicallyrelevant concentration that was used for maintaining the cells in culture. (**D**) Quantification of GR and β-catenin from immunoblots normalized to β-actin. Data include 5 independent sample sets and are represented as mean ± SEM. (**E**) Total GR expression in the three pairs of cell lines (sensitive vs. DR) was detected in the same blots. (**F**) Quantification of GR from immunoblots relative to GAPDH. Data include at least 3 independent immunoblots and are represented as mean ± SEM (* *p* < 0.05, ** p < 0.01, *** p < 0.001).

Using a panel of PCa cell lines grown in standard cell culture media supplemented with fetal bovine serum (FBS) (which contains glucocorticoids), we observed by immunoblotting, using non-cross-reactive specific antibodies, that the total intracellular AR and GR expression levels are inversely related. In contrast, total β-catenin expression levels did not vary relative to these proteins (Figure 1A,B). To determine the effect of GR signaling in chemoresistant PCa, we generated DTX-resistant (DR) sublines of the 22rv1 (AR+/GR+), PC3 (AR−/GR+), and DU145 (AR−/GR+) cell lines. These DTX-resistant cell lines showed IC50 fold increases of 47.0 (22rv1-DR), 24.8 (PC3-DR), and 32 (DU145-DR) in the presence of DTX, compared to their parental drug-sensitive cell lines (Figure S1A–C). The most significant difference in growth rates between sensitive and resistant cell lines was observed at 10 nM DTX given that this was the endpoint drug concentration used to select resistant cells. All DTX-resistant cell lines exhibited MDR1/ABCB1 expression (Figure S1D,E). The resistant phenotype was also validated in 22rv1 cells by the elevated expression (2.2-fold) of AR-V7 in 22rv1-DR cells compared to the parental cells (Figure S1F). This is consistent with a previous report that AR-V7 expression increases after chronic DTX exposure [52]. In addition, 22rv1-DR and PC3-DR cells grown under standardcell culture media supplemented with FBS showed increased total GR and β-catenin expression compared to their corresponding DTX-sensitive cell lines, whereas DU145-DR cells expressed lower total GR and β-catenin levels than their DTX-sensitive counterparts (Figure 1C,D).

The immunoblots for the three cell line pairs (sensitive vs. DR) used for this quantification were processed separately for each pair (Figure 1C), and GR protein expression was then normalized to β-actin (Figure 1D). To confirm that GR is consistently expressed at higher levels in DTX-sensitive DU145 cells compared to 22rv1 and PC3 cells, we performed a separate experiment in which the GR levels in the three cell line pairs were compared side by side in the same blots (Figure 1E). The expression levels of GR were then determined relative to GAPDH (Figure 1F). The results showed higher total protein expression of GR in the drug-sensitive DU145 cells compared to 22rv1 and PC3 cells, with lower expression in DU145-DR cells compared to 22rv1-DR and PC3-DR cells.

### 2.2. Glucocorticoid-Induced GR Nuclear Translocation in DTX-Sensitive and -Resistant PCa Cells

**Figure 2:**
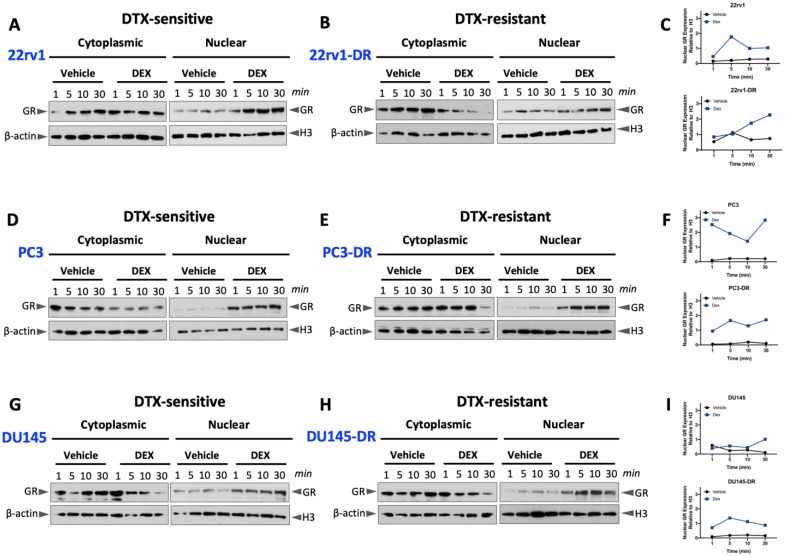
GR nuclear translocation in DTX-sensitive and DTX-resistant PCa cells in response to dexamethasone. DTX-sensitive and DTX-resistant 22rv1 (**A**,**B**), PC3 (**D**,**E**), and DU145 (**G**,**H**) cells were cultured in medium depleted of glucocorticoids (containing CS-FBS) for 12 h prior to treatment with 10 nM dexamethasone for 1, 5, 10, or 30 min, and subcellular cytoplasmic and nuclear proteins were extracted. The blots included in this figure were quantified to show nuclear GR expression in dexamethasone-treated cells (blue squares) compared to vehicle-treated cells (black circles) (**C**,**F**,**I**). GR and the loading controls β-actin (cytoplasmic) and histone H3 (nuclear) were detected by immunoblotting.

As a first step in improving our understanding of GR function in DTX-resistant PCa cells, we examined GR nuclear translocation in these cells and their DTX-sensitive counterparts.As expected, all DTX-sensitive and -resistant cell lines exhibited higher levels of GR nuclear expression in response to 10 nM dexamethasone, compared to control vehicle-treated cells. These experiments were performed with cells grown in media supplemented with charcoal-stripped FBS (CS-FBS) to remove serum glucocorticoids (Figure 2A–I). GR nuclear translocation was relatively rapid, with nuclear accumulation evident in all cell lines within one minute. We did not observe significant differences in GR nuclear translocation between the DTX-sensitive and DTX-resistant cell lines. These results confirmed the increased nuclear accumulation of GR in PCa cell lines exposed to dexamethasone. In addition, they showed that, despite the lower basal expression levels of this protein in DU145-DR cells compared to their DTX-sensitive counterparts (Figure 1C), its glucocorticoid-induced nuclear accumulation in both DTX-sensitive and DTX-resistant cells was relatively rapid and efficient (Figure 2G,H). For these experiments, histone H3 was used as a marker for the nuclear fractions and was not detected in the cytoplasmic fractions (Figure S2). We should note that due to the large number of blots, cellular fractions, and time points used to generate Figure 2, it was not possible to repeat all the blots sufficiently to achieve a statistically significant quantification of protein expression.

### 2.3. Dexamethasone Induces Nuclear β-Catenin Accumulation in DTX-Resistant PCa Cells

**Figure 3:**
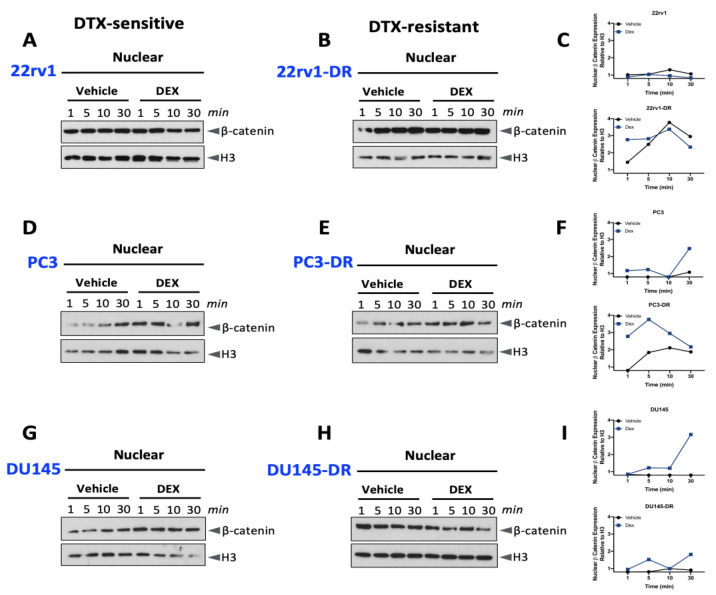
Total β-catenin nuclear translocation in DTX-sensitive and DTX-resistant PCa cells after dexamethasone treatment. Following growth for 12 h in medium containing CS- FBS, DTX-sensitive, and DTX-resistant 22rv1, PC3, and DU145 cells were treated with 10 nM dexamethasone for the indicated times, and soluble nuclear proteins were extracted for immunoblotting detection of GR and β-catenin in (**A**) 22rv1, (**B**) 22rv1-DR, (**D**) PC3, (**E**) PC3-DR, (**G**) DU145, and (**H**) DU145-DR cells. The blots included in this figure were quantified to show nuclear β-catenin expression in dexamethasone-treated cells (blue squares) compared to vehicle-treated cells (black circles) (**C**,**F**,**I**). Histone H3 was used as a marker for nuclear fractions.

To determine whether glucocorticoid-induced GR nuclear translocation also affects β-catenin nuclear translocation, we measured the nuclear accumulation of total β-catenin in PCa cells grown in CS-FBS following treatment with 10 nM dexamethasone. Unlike GR nuclear translocation, we did not detect a clearly distinct increase in glucocorticoid-dependent nuclear levels of total β-catenin, compared to the vehicle controls, in the six cell lines (Figure 3). There seemed to be a slight increase in total β-catenin expression in the nuclei of PC3, PC3-DR, and DU145 cells treated with dexamethasone compared to vehicle controls (Figure 3D–G,I), but, as noted above for Figure 2, due to a large number of blots and samples, it was not possible to repeat all the blots sufficient times to achieve a statistically significant quantification of protein expression.

Given that in these experiments we used an antibody that recognizes total β-catenin, we investigated whether active β-catenin was present in the nucleus of DTX-resistant cells treated with dexamethasone. To evaluate this, we used an anti-β-catenin monoclonal antibody that recognizes this protein’s non-phosphorylated, active form. Phosphorylation of β-catenin in the cytoplasm targets this protein for ubiquitination and proteasomal degradation in the destruction complex, whereas non-phosphorylated β-catenin accumulates in the cytoplasm for subsequent translocation into the nucleus [35].

**Figure 4:**
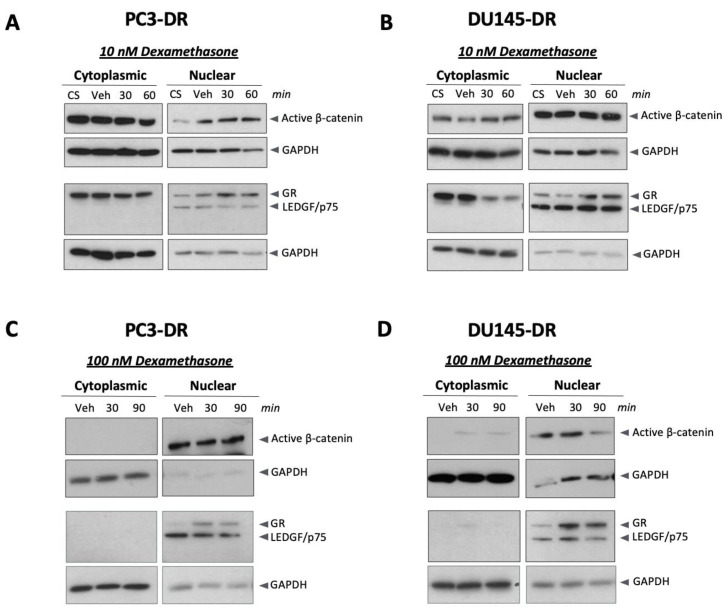
Nuclear localization of active β-catenin in DTX-resistant prostate cancer cells after dexamethasone treatment. Cells grown for 12 h in medium containing CS-FBS were then treated with 10 nM dexamethasone for the indicated times, and cytoplasmic and nuclear proteins were extracted for immunoblotting detection of GR and non-phosphorylated active β-catenin in (**A**) PC3-DR and (**B**) DU145-DR cells. A similar experiment was conducted using 100 nM dexamethasone in (**C**) PC3-DR and (**D**) DU145-DR cells. For these experiments, LEDGF/p75 and GAPDH were used as markers for nuclear and cytoplasmic fractions, respectively. CS—charcoal-stripped medium control; Veh—vehicle (ethanol) control. Blots are representative of 2 independent experiments.

We detected active β-catenin in nuclear and cytoplasmic fractions of PC3-DR and DU145-DR cells treated with 10 nM dexamethasone (Figure 4A,B) and in the controls. This suggested that dexamethasone did not exert a major influence on β-catenin nuclear translocation at this concentration. However, in the presence of 100 nM dexamethasone, a higher concentration that promoted a robust GR nuclear translocation in DTX-resistant cells, GR and active β-catenin were present mostly in the nucleus in both cell lines (Figure 4C,D). Thepurity of the subcellular fractions was monitored with LEDGF/p75, a nuclear oncoprotein associated with DTX-resistance and GR signaling [27,53,54,55], and GAPDH, a cytoplasmic enzyme that in cells stressed by pro-apoptotic stimuli such as cytotoxic drugs can also translocate into the nucleus [56]. However, LEDGF/p75 expression was detected exclusively in the nuclear fractions and was not influenced by dexamethasone during the indicated treatment periods. Moreover, GAPDH was abundant in cytoplasmic fractions with diminished levels in nuclear fractions (Figure 4A–D). These results suggested that exposure of PC3-DR and DU145-DR cells to non-cytotoxic, high concentrations of dexamethasone leads to a more efficient nuclear translocation of active β-catenin.

### 2.4. GR and β-Catenin Interact in PCa Cells

**Figure 5:**
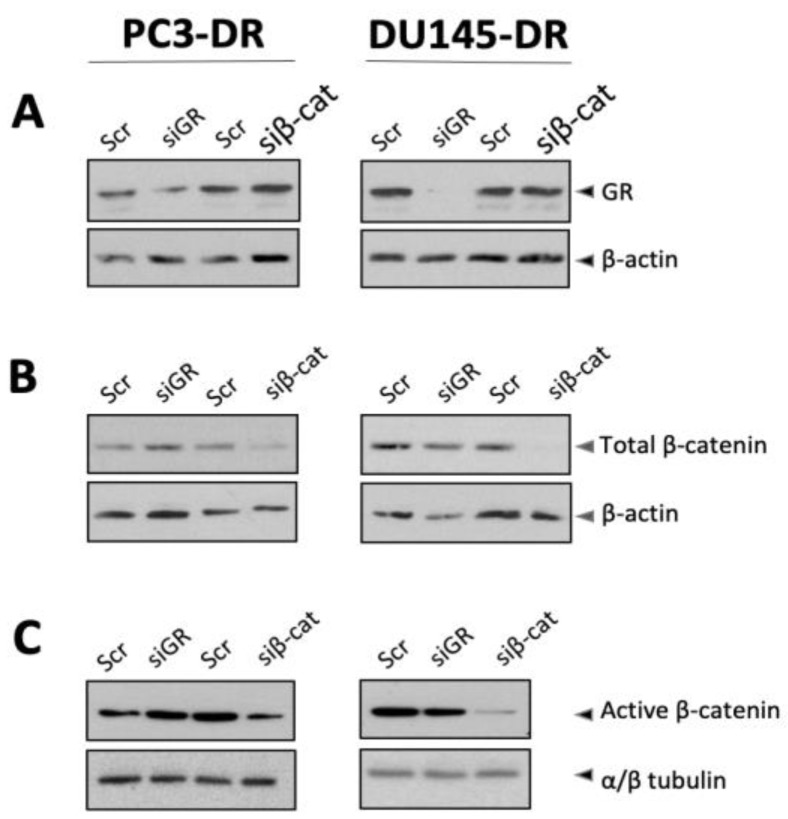
The expression of GR and β-catenin in DTX-resistant PCa cells is not inter-dependent. siRNA-mediated knockdown of GR or β-catenin was achieved in PC3-DR and DU145-DR using 100 nM each of non-specific scrambled oligo (Scr), siRNAs specific for GR (siGR), or siRNAs specific for β-catenin (siβ-cat) for 24 h. Knockdowns were detected by immunoblotting using specific antibodies for GR (**A**), total β-catenin (**B**), and active β-catenin (**C**) in PC3-DR and DU145-DR lysates. β-actin and αβ-tubulin were used as loading controls.

As mentioned previously, the structural and functional similarities between GR and AR (an interacting partner of β-catenin) led us to determine, using co-immunoprecipitation (co-IP) studies with specific antibodies, if GR and β-catenin are part of an interacting nuclear complex in PCa cells. Prior to performing these experiments, we first determined (1) if the expression levels of GR and β-catenin in PCa cells are inter-dependent and (2) if there is any in silico evidence of a potential interaction between these two proteins. Thus, we performed individual siRNA-mediated knockdown of GR (94 kDa) and β-catenin (92 kDa). As expected, depletion of GR in both PC3-DR and DU145-DR cells led to downregulation of GR expression levels (Figure 5A); however, the levels of β-catenin did not seem to be affected (Figure 5B). Similarly, depletion of β-catenin downregulated the expression levels of this protein (Figure 5B) and its active form (Figure 5C) but did not appear to affect GR levels (Figure 5A). These results suggested that GR and β-catenin do not regulate each other in DTX-resistant cells since depletion of one protein did not affect the expression of the other. They also validated the specificity of the GR and β-catenin antibodies used in subsequent co-IP experiments.

**Figure 6:**
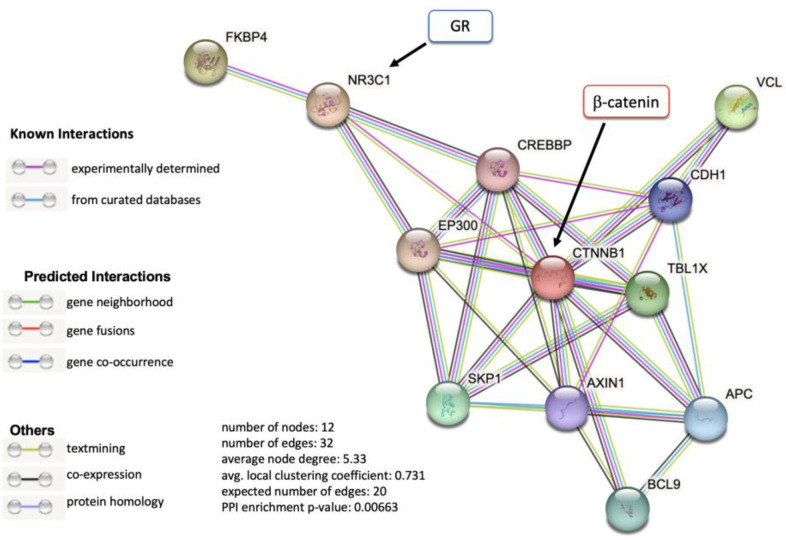
GR and β-catenin interaction module detected by STRING analysis. GR is encoded by the *NR3C1* gene and β-catenin by the *CTNNB1* gene. The only GR/β-catenin interactions detected in this analysis were from gene fusion (red line) and text mining (light green).

As a first step in exploring a possible interaction between GR (encoded by the *NR3C1* gene) and β-catenin (encoded by the *CTNNB1* gene), we performed a bioinformatic inquiry using the STRING platform for protein–protein interaction networks and functional enrichment analysis. This analysis revealed that GR and β-catenin have overlapping interactors such as EP300 and CREBBP (Figure 6). The interactions between GR and β-catenin identified in the STRING analysis were from gene fusions (individual gene fusion events in the genome of the same species) and text mining (statistically relevant co-occurrence of the gene names in the literature). This analysis, however, did not reveal a GR/β-catenin interaction that was previously experimentally determined.

**Figure 7:**
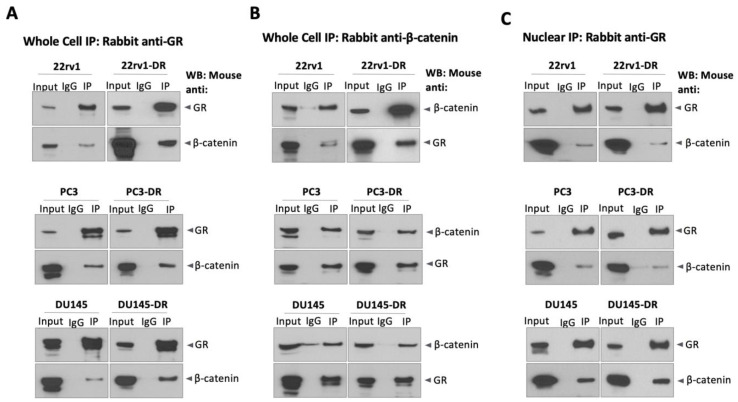
GR and β-catenin interact in prostate cancer cells. Co-immunoprecipitation studies were performed in soluble protein lysates from DTX-sensitive and DTX-resistant 22rv1, PC3, and DU145 cells cultured in complete medium. Rabbit monoclonal antibodies specific for GR (**A**) or total β-catenin (**B**) were used for protein immunoprecipitation, and both proteins were detected by immunoblotting using specific mouse monoclonal antibodies. Co-immunoprecipitation of GR and β-catenin was also performed using nuclear lysates from DTX-sensitive and DTX-resistant prostate cancer cells (**C**). Prior to immunoprecipitation, cells were cultured in medium supplemented with CS-FBS for 12 h and subsequently treated with 10 nM dexamethasone for 30 min, followed by isolation of nuclear protein complexes. Immunoprecipitation of nuclear proteins was also performed using rabbit antibodies specific for GR and β-catenin, followed by immunoblotting detection of both proteins using specific mouse monoclonal antibodies. Normal rabbit IgG was used as a negative control for immunoprecipitation, and whole cell lysates collected concomitantly with immunoprecipitation reactions were used as inputs (10% of IP). Immunoprecipitations were performed at least 3 times independently, and representative blots are shown.

To experimentally determine if GR and β-catenin interact in PCa cells, we performed co-IP studies. The results revealed that endogenous GR and β-catenin interact in both DTX-sensitive and DTX-resistant cells, as β-catenin was detected in GR immunoprecipitates from three different PCa cell line pairs (Figure 7A). These results were confirmed by reverse co-IP using β-catenin-specific antibodies (Figure 7B). To determine if the GR/β-catenin interaction occurs in the nucleus, we also performed co-IPs using nuclear extracts. As low GR levels were generally detected in the nuclei of unstimulated cells (Figure 2), we performed the nuclear co-IPs following treatment with 10 nM dexamethasone for 30 min. β-catenin was detectable in a nuclear complex with GR regardless of DTX-resistance status (Figure 7C). These results showed that GR and β-catenin interact in PCa cells, forming a complex that can be detected in the nucleus and is not dependent on DTX resistance.

### 2.5. Co-Inhibition of GR and β-Catenin Resensitizes DTX-Resistant PCa Cells to DTX-Induced Cytotoxicity

**Figure 8:**
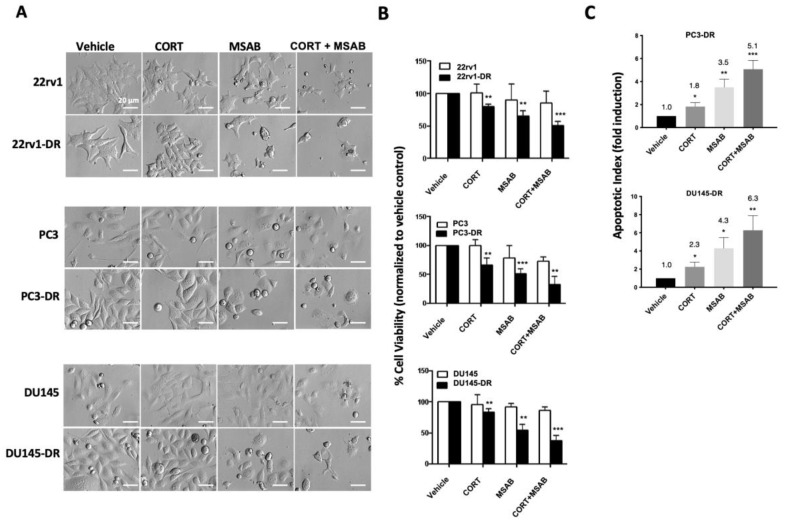
Co-inhibition of GR and β-catenin re-sensitizes DTX-resistant PCa cells to docetaxel. (**A**) Hoffman modulation microscopy (800×) images of DTX-sensitive and DTX-resistant 22rv1, PC3, and DU145 cells treated with CORT-108297 (CORT, 1 μM), MSAB (1 μM), or CORT-108297 plus MSAB (CORT + MSAB, 1 μM each) for 72 h in adherent cultures. (**B**) Cell viability following treatments was assessed using MTT assays. DMSO was used for vehicle control. (**C**) Apoptotic index (fold induction of apoptosis) was determined by flow cytometry using Annexin V and 7AAD for PC3-DR and DU145-DR cells treated with the GR and β-catenin inhibitors**.** DTX-resistant cells, but not sensitive cells, were cultured throughout the treatment in medium containing 10 nM DTX. All treatments were done in cells cultured in media supplemented with CS-FBS and 10 nM dexamethasone. Data are representative from 3 independent experiments and represented as mean ± SEM. * *p* < 0.05, ** *p* < 0.01, *** *p* < 0.001. The scale bar for all images is set at 20 μm.

To determine the functional relevance of the GR/β-catenin interaction to DTX resistance, we evaluated the effects of inhibiting these two proteins, individually or combined, on the viability of DTX-sensitive and DTX-resistant cells. Inhibition of β-catenin was achieved with MSAB (methyl 3-{[(4-methylphenyl)sulfonyl]amino}benzoate), a selective and potent small molecule inhibitor of the Wnt/β-catenin pathway that binds to β-catenin and promotes its degradation, downregulating target genes of this pathway [57]. For GR inhibition, we used the selective GR modulator CORT-108297 (hereafter referred to as CORT) [58]. DTX-sensitive and DTX-resistant PC3, DU145, and 22rv1 cells were grown for 12 h in medium supplemented with CS-FBS and then treated for 72 h with MSAB (1 μM) alone, CORT-108297 alone (1 μM), or in combination (CORT + MSAB) in the presence of 10 nM dexamethasone but the absence of DTX. These treatments had non-significant effects on the viability of the DTX-sensitive cell lines (measured by MTT assays) compared to the corresponding vehicle control (Figure 8A,B). However, the treatments significantly decreased the viability of the DTX-resistant cells in the presence of 10 nM DTX (maintenance concentration) compared to the corresponding vehicle control (Figure 8B). There was a significant decrease in cell viability with both individual and co-inhibition of GR and β-catenin compared to vehicle controls in the DTX-resistant PCa cell lines, with CORT + MSAB exerting the most potent inhibitory effects, followed by MSAB alone and CORT alone (Figure 8B). We also measured the apoptotic index induced by the treatments in DTX-resistant cells, observing significant fold-induction of apoptosis in PC3-DR and DU145-DR cells treated with CORT + MSAB (5.1-fold and 6.3-fold, respectively), MSAB (3.5-fold and 4.3-fold, respectively) and CORT (1.8-fold and 2.3-fold, respectively) (Figure 8C). We could not determine the apoptotic index in 22rv1-DR cells due to their excessive aggregation during the flow cytometry analysis. The observed results indicated that while exposure of DTX-sensitive PCa cells to GR and β-catenin inhibition had minimal impact on their viability, inhibition of these two proteins in DTX-resistant cells in the presence of DTX and dexamethasone had a potent negative effect on cell survival. This suggested a dependence on GR and β-catenin in DTX-resistant, but not DTX-sensitive, PCa cells.

### 2.6. Co-Inhibition of GR and β-Catenin Reduces Tumorsphere Formation and Stemness in DTX-Resistant PCa Cells

**Figure 9:**
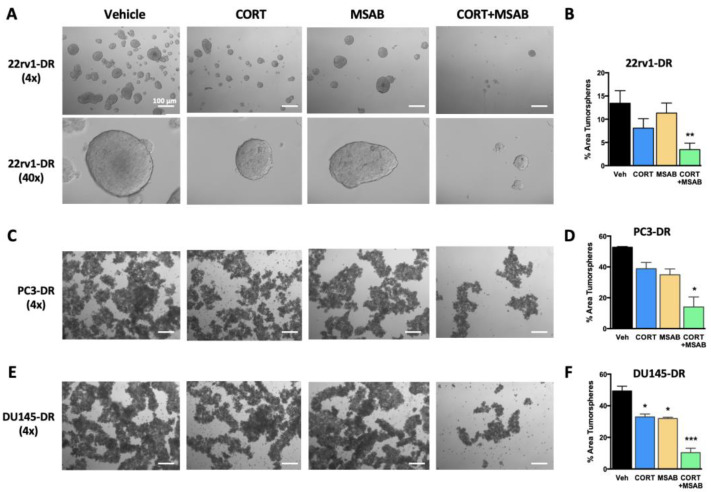
Co-inhibition of GR and β-catenin impairs docetaxel-resistant prostate tumorsphere formation. DTX-resistant 22rv1-DR (**A**), PC3-DR (**C**), and DU145-DR (**E**) cells were cultured as tumorspheres in ultra-low adherence dishes in Tumorsphere XF medium containing 10 nM DTX and vehicle (DMSO), CORT-108297 (CORT, 1 μM), MSAB (1 μM), or CORT-108297 plus MSAB (CORT + MSAB, 1 μM each) for 6 days and imaged using an Olympus IX70 microscope equipped with Hoffman modulation. Images were acquired at 4× or 40× magnification. The scale bar for all images (4× magnification) is set at 100 μm. Tumorsphere area was quantified from quadruplicate images for each cell line (**B**,**D**,**F**). Data are representative from 3 independent experiments and are represented as mean ± SEM. * *p* < 0.05, ** *p* < 0.01, *** *p* < 0.001.

Next, we assessed the contribution of GR and β-catenin signaling to the stemness properties of tumorspheres derived from DTX-resistant PCa cells. We previously demonstrated that PC3-DR and DU145-DR cells exhibit a transcriptomic program associated with stemness, display CSC markers, and show enhanced tumorsphere formation capacity compared to DTX-sensitive cells [25]. Co-inhibition of GR and β-catenin with CORT + MSAB, but not treatment with individual inhibitors, significantly impaired tumorsphere formation in 22rv1-DR and PC3-DR cells (Figure 9A–D), whereas both individual and co-inhibitory treatments impaired tumorsphere formation in DU145-DR (Figure 9E,F). The most profound effects were observed with CORT + MSAB (Figure 9B,D,F).

**Figure 10:**
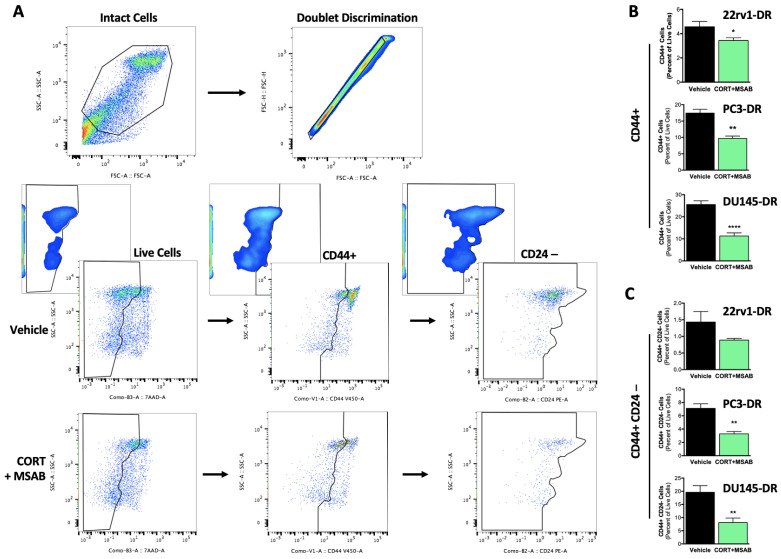
Co-inhibition of GR and β-catenin reduces the percent of cells with stemness markers in docetaxel-resistant tumorspheres. (**A**) Post-acquisition gating strategy, shown in a representative PC3-DR analysis. Data are depicted as pseudo color plots that denote population densities (high: red/orange, intermediate: yellow, low: blue/green) in bivariate settings (X-Y plane). The color densities do not refer to the spectral emission of the cells depicted, but rather the density of cells relative to one another. Doublet discrimination and fluorescence-minus-one controls (graph insets) are depicted with smoothing to better visualize the gating strategy. (**B**) CD44+ expression presented as percent of live (7AAD-negative) cells staining positive for CD44 in 22rv1-DR, PC3-DR, and DU145-DR cells from tumorspheres treated with CORT-108297 plus MSAB (CORT + MSAB, 1 μM each) compared to vehicle (DMSO) control. (**C**) CD44+ CD24– expression presented as percent of live cells staining positive for CD44 and negative for CD24 in 22rv1-DR, PC3-DR, and DU145-DR cells from tumorspheres treated with CORT-108297 plus MSAB (CORT + MSAB, 1 μM each) compared with vehicle control. Data are representative from 3 independent experiments and are represented as mean ± SEM. * *p* < 0.05, ** *p* < 0.01, **** *p* < 0.0001.

The co-inhibition of GR and β-catenin significantly reduced the proportion of cells with CSC-like properties within tumorspheres, assessed by flow cytometric detection of CSC-like populations based on CD44 and CD24 status (Figure 10A). This was evident by a significant reduction in the percent of CD44+ cells in tumorspheres from 22rv1-DR, PC3-DR, and DU145-DR cells treated with CORT + MSAB compared with controls (Figure 10B). Further data stratification revealed a significant reduction in CD44+ CD24– cells in tumorspheres from PC3-DR and DU145-DR cells treated with CORT + MSAB and a trending decrease, albeit not significant, in tumorspheres from 22rv1-DR cells treated with CORT + MSAB (Figure 10C). Together, these results indicated that GR and β-catenin contribute to stemness and tumorsphere formation in DTX-resistant PCa cells.

A previous study characterized a novel enhancer at the GR locus marked by acetylated H3K27ac that is required for GR upregulation in enzalutamide-resistant cells [13]. To determine if the BET-bromodomain-containing family of chromatin regulators, which bind acetylated lysines at enhancers to regulate gene expression, are involved in regulating GR expression in this context, the authors of that study treated enzalutamide-resistant cells with the BRD4 inhibitor JQ1. They observed a dose-dependent (0.01–1 μM) decrease in the expression of GR and some of its target genes [13]. We observed that treatment with 1 μM JQ1 significantly inhibited tumorsphere formation in both PC3-DR and DU145-DR cells (Figure S3A–D). In addition, JQ1 also drastically reduced the percent of CD44+ and CD44+ CD24– cells in the DTX-resistant tumorspheres (Figure S3E,F). These results indicated that blocking BET-bromodomain proteins, which regulate GR expression among other genes [13], reduces tumorsphere formation and stemness in DTX-resistant cells, consistent with our results from the co-inhibition of GR and β-catenin.

## 3. Discussion

The contribution of GR signaling to ARSI resistance in mCRPC is well-established, and some of the underlying mechanisms have been recently uncovered [7,8,9,10,11,12,13,14,15,16,17,18,19,20,21,59]. However, while the role of GR signaling in promoting chemoresistance has been suggested for other solid tumors [60,61,62], its role in mCRPC chemoresistance just recently emerged [16,22,27]. The dual role of GR signaling in promoting resistance to both ARSI and taxane chemotherapy during the treatment of mCRPC implicates this nuclear receptor as a key player in PCa therapy cross-resistance, a common occurrence in which a pre-existing or acquired mechanism that promotes resistance to a particular drug treatment (e.g., ARSI) results in resistance to a subsequent drug or therapy (e.g., taxane chemotherapy) [6,63]. Therapy cross-resistance has implications for determining the safest and more effective sequence in which different drugs should be administered during PCa progression [6]. Further, establishing the contribution of GR signaling to PCa therapy cross-resistance is highly relevant clinically given that both ARSI and taxane chemotherapy are administered to patients with mCRPC concomitantly with glucocorticoids (e.g., dexamethasone, prednisone, or prednisolone) to ameliorate the side effects of the drugs and make the chemotherapeutic regimens more tolerable [64,65]. However, a potentially negative outcome of glucocorticoid administration is the likelihood of an increased activation of GR signaling, which may consequently lead to therapy resistance.

A previous study demonstrated increased GR expression in tumors from PCa patients treated with neoadjuvant chemotherapy and observed GR overexpression in the DTX-resistant cell lines PC3-DR, DU145-DR, and 22rv1-DR [22]. Our studies confirmed this observation for the PC3-DR and 22rv1-DR cell lines; however, our DU145-DR cells consistently exhibited reduced GR expression compared to the sensitive DU145 cells. While these divergent observations could be explained by differences in the experimental conditions and the batches of DU145 cell lines used, we could also speculate that GR downregulation in DU145-DR cells may result from a negative feedback mechanism activated when the parental DU145 cells, which express high basal GR levels, befall under selective pressure to further increase GR signaling in the presence of DTX and culture medium glucocorticoids. Consistent with this, an earlier study reported higher GR levels in DU145 cells compared to PC3 cells and that dexamethasone decreased GR expression and DU145 proliferation and xenograft growth [66]. Despite the decrease in GR expression consistently observed in our DU145-DR cells compared to their sensitive counterparts, there was still a noticeable rapid increase in translocated nuclear GR in these cells after dexamethasone treatment compared to vehicle controls (Figure 2F).

GR antagonism in DTX-resistant PCa cell lines with mifepristone (RU-486) in the presence of DTX resulted in downregulation of the anti-apoptotic proteins Bcl-2 and Bcl-xL, implicating regulation of apoptosis as a potential mechanism by which GR promotes DTX resistance [22]. Consistent with these results, our studies targeting GR and β-catenin with individual or combined small molecule inhibitors (CORT and MSAB) in chemoresistant PCa cells in the presence of DTX showed an increase in the apoptotic index of treated cells (Figure 7C). Of note, the induction of apoptosis was more robust with MSAB alone or with the CORT + MSAB combination, implicating β-catenin as a key player in promoting DTX resistance. In addition to regulating pro-apoptotic proteins, GR may promote DTX resistance by upregulating the expression of stress response oncoproteins such as LEDGF/p75, a transcription co-activator, and Clusterin, a chaperone that inhibits protein aggregation [27]. In a previousstudy, our group demonstrated that LEDGF/p75 and several members of its protein interactome, which include the JPO2-cMYC and Menin-MLL oncogenic complexes, promote the survival, clonogenic growth and tumorsphere formation of PC3-DR and DU145-DR cells [55]. Acting as a transcription co-activator, LEDGF/p75 interacts with several oncogenic transcription factors to tether them to active chromatin sites and facilitate RNA polymerase II transcription [67,68]. An interesting observation in our previous study was that the nuclear co-localization and interaction of LEDGF/p75 with JPO2 was dependent on GR-mediated nuclear translocation of JPO2, implicating GR not only as a regulator of LEDGF/p75 expression but also a potential component of its interactome [55]. Ongoing studies by our group are investigating how the interplay between GR, LEDGF/p75, and members of their protein interactome contribute to DTX resistance in PCa.

GR expression is reduced in primary PCa compared with normal prostate tissues but restored in metastatic tissues [14] and upregulated in anti-AR therapy-resistant and chemotherapy-treated tissues [22,45]. Consistent with this, we explored the cancer genome atlas (TCGA) database to determine a possible expression correlation between GR and β-catenin in PCa samples. However, we could not detect GR expression, possibly because of the overrepresentation of prostatectomy-derived primary PCa tissues in this database. β-catenin expression correlates with increasing Gleason score, metastasis, resistance to anti-androgen therapy, and chemoresistance [34,42,44]. Upregulation of GR and β-catenin in mCRPC proceeds despite the declining influence of AR signaling, particularly in neuroendocrine differentiated PCa, characterized by AR loss and resistance to anti-AR therapy [69,70]. Due to their neuroendocrine features and lack of AR expression, PC3 and DU145 cells are thought to represent this advanced PCa stage [71]. Prostate CSCs also lack AR [72,73], supporting their involvement in therapy resistance.

Our hypothesis that the GR and β-catenin interaction may contribute to stemness and DTX resistance originated from previous observations that AR and GR share structural similarities and transcriptomic profiles [11,12,51]. Furthermore, β-catenin interacts with AR to promote its transcriptional activity [30,31,32,33], and GR and Wnt/β-catenin signaling promote tumorsphere formation and stemness in PCa cells [14,74]. To evaluate the role of the GR/β-catenin interaction in DTX-resistant cells compared with DTX-sensitive cells, we first performed nuclear translocation studies following GR activation with dexamethasone in a subacute time frame. All the cell lines displayed a rapid accumulation of nuclear GR in the presence of dexamethasone, compared to vehicle controls, suggesting that this accumulation may not be dependent on taxane resistance.

To evaluate if glucocorticoid signaling promotes β-catenin nuclear translocation, we assessed nuclear β-catenin levels (total and active) following treatment with dexamethasone. Of note, a prior study using GR-overexpressing PC3 cells demonstrated limited nuclear β-catenin accumulation following dexamethasone treatment [31], a finding we replicated with PC3 cells expressing endogenous GR. In addition, we observed that both PC3-DR and DU145-DR cells treated with 10 nM dexamethasone showed active β-catenin in the nucleus, although this protein also accumulated in the cytoplasm. Yet, in the presence of 100 nM dexamethasone, both active β-catenin and GR localized almost exclusively in the nucleus. Excess of glucocorticoids is known to modulate the expression of Wnt signaling inhibitors in osteosarcoma cells, influencing the cytoplasmic accumulation and nuclear translocation of β-catenin [75]. However, it is plausible that in DTX-resistant cells, the robust nuclear GR translocation induced by 100 nM dexamethasone may facilitate β-catenin translocation if both proteins are in a complex. Furthermore, the glucocorticoid-mediated nuclear translocation of β-catenin is consistent with the observed translocation of this protein to the nucleus in the presence of androgens to enhance AR transcription [31]. Further studies are needed to determine if the glucocorticoid-mediated nuclear translocation of β-catenin enhances GR signaling, particularly in the context of PCa chemoresistance.

Previous reports of GR/β-catenin interaction have been limited to findings in yeast-2-hybrid systems and U2OS osteosarcoma cells, where GR suppresses the expression of downstream β-catenin target genes [76]. It is likely that the overall role of the GR/β-catenin interaction differs between tissue and tumor cell types. We detected the GR/β-catenin interaction in all the examined PCa cell lines, including DTX-resistant cells. This suggests that this interaction is relevant in advanced PCa and not exclusive to cells that developed taxane resistance. Although we did not determine in our study the binding sites within GR and β-catenin mediating this interaction, we could speculate that the C-terminal ligand binding domain (LBD) and nuclear localization signals within the GR sequence may play a role, as similar sequences in the AR protein have been implicated in binding to β-catenin [32]. It is also possible that post-translational modifications (PTMs) may play a role in mediating this interaction. However, little is known about PTM-mediated GR activity in the context of tumor progression and therapy resistance inPCa. In the context of GR and β-catenin interactions, if the interaction interface mirrors that of AR with β-catenin or the previously described GR–β-catenin interaction in osteosarcoma cells, PTMs in the DNA-binding domain (DBD; residues 421-486) may influence the interaction with help from the LBD (residues 528-777 for GR) [31,76]. Notably, none of the 22 currently known phosphorylation sites on the GR protein are located within the DBD or LBD (https://www.uniprot.org/uniprotkb/P04150/entry#ptm_processing (accessed on 12 March 2023)). However, this does not rule out possible allosteric effects of phosphorylation or PTMs outside these regions and subsequent recruitment of other binding partners that may facilitate the recruitment of β-catenin to the GR into a multifactorial complex. Further work is needed to understand the tissue and context-specific roles of PTMs in prostate tumor aggressiveness, therapy response, and disease progression.

Combinatorial treatment with CORT-108297 plus MSAB re-sensitized DTX-resistant cells and tumorspheres to DTX and reduced the frequency of CD44+ and CD44+ CD24– populations within tumorspheres, suggesting that co-inhibition of GR and β-catenin could be a promising therapeutic strategy for chemoresistant CRPC. However, a limitation of our study is that CORT-108297, while being a selective GR modulator, may not be a true antagonist. Nevertheless, our complementary approach using the BRD4 inhibitor JQ1, which blocks enhancer-mediated upregulation of GR [13], supported a role for GR in promoting tumorsphere formation and stemness in PC3-DR and DU145-DR cells. It should be noted, however, that BRD4 binding was not detected at the GR enhancer, suggesting that JQ1 may target different proteins responsible for GR expression [13]. Given the multiple roles of the BET bromodomain family in regulating the expression of many genes other than GR, we cannot rule out that the JQ1 inhibitory effects we observed on tumorsphere formation and stemness may not be GR-related and instead could be mediated by other protein targets of this family. JQ1, combined with DTX, was recently reported to inhibit LNCaP (AR+) tumorsphere formation at higher levels than JQ1 or DTX alone [77]. While PC3-DR and DU145-DR cells are AR negative, the contribution of other BET bromodomain-regulated proteins should be considered. Future studies with more selective GR modulators, such as relacolirant, currently in clinical trials to determine its effectiveness in promoting taxane responses in solid tumors [78], should shed additional mechanistic insights into the contribution of GR to taxane resistance.

Although the contribution of Wnt/β-catenin to PCa stemness is well established [33,46,79], knowledge of mechanisms underlying its role in chemoresistance is still limited. Recently, PC3 and DU145 cells with active Wnt/β-catenin signaling exhibited CSC properties, higher resistance to DTX, and suppression of H3K27 trimethylation, contributing to PCa heterogeneity [80]. Wnt/β-catenin signaling was also reported to be activated by the E3 ubiquitin ligase EDD in DU145 and 22rv1 cells, leading to increased DTX resistance [36]. Further, the addition of the tankyrase inhibitor XAV939, an antagonist of Wnt/β-catenin signaling, in combination with DTX in PC3 cells led to the suppression of microtubule-mediated cell division processes linked to taxane resistance [38]. Moreover, the observation that chemotherapy-induced leukemia senescence gives rise to therapy-resistant CSCs via a mechanism that involves robust β-catenin upregulation implicates Wnt/β-catenin signaling in a cellular reprogramming that promotes cancer stemness and chemoresistance [81]. PCa cellular reprogramming in response to chronic DTX exposure will likely give rise to a cell population with increased Wnt/β-catenin signaling, resulting in the sustained growth of therapy-resistant CSC cells.

While the Wnt/β-catenin and GR pathways can mutually inhibit each other in non-tumor tissues and cells [75], our results suggest that in PCa cells, they may cooperate via protein–protein interactions to promote stemness and drug resistance. The recent finding that Wnt/β-catenin signaling is differentially activated between different racial/ethnic groups also underscores the importance of mapping therapy resistance patterns across diverse populations to enable precision medicine-based advancements in prognosis prediction and therapeutic selection [82]. Future follow-up studies should include racially/ethnically diverse mouse xenografts models (derived from both DTX-resistant PCa cell lines and DTX-resistant patient tumors [PDX]) treated with combinations of novel selective GR modulators (i.e., exicorilant and relacorilant), β-catenin inhibitors (MSAB) and DTX, to validate our in vitro results implicating the GR/β-catenin axis in PCa taxane resistance in diverse pre-clinical in vivo models.

## 4. Materials and Methods

### 4.1. Cell Culture

The following PCa cell lines were purchased from the American Type Culture Collection (ATCC, Manassas, VA, USA): LNCaP (CRL-1740), VCaP (CRL-2876), MDA PCa 2b (CRL-2422), 22rv1 (CRL-2505), PC3 (CRL-1435) and DU145 (HTB-81). LNCaP, PC3, DU145, and 22rv1 cells were cultured in RPMI 1640 medium (Corning, Glendale, AZ, USA, Cat# 10040CV) supplemented with 10% fetal bovine serum (FBS) (Corning, Glendale, AZ, USA, Cat# 35010CV) and Penicillin/Streptomycin (Corning, Glendale, AZ, USA, Cat# 30002CI). MDA PCa 2b cells were cultured in F12K medium (Corning, Glendale, AZ, USA, Cat# 10-025-CV) supplemented with 20% FBS and 25 ng/mL cholera toxin (Sigma-Aldrich, St. Louis, MO, USA, Cat# 8052), 10 ng/mL human epidermal growth factor (Sigma-Aldrich, St. Louis, MO, USA, Cat# E9644), 5 ug/mL insulin (Sigma-Aldrich, St. Louis, MO, USA, Cat# I0516), 5.8 ng/mL selenous acid (Sigma-Aldrich, St. Louis, MO, USA, Cat# 229857), 100 pg/mL hydrocortisone (Sigma-Aldrich, St. Louis, MO, USA, Cat# H0135), and 700 ng/mL *O*-phosphorylethanolamine (Sigma-Aldrich, St. Louis, MO, USA, Cat# P0503). VCaP cells were cultured in DMEM (ATCC, Manassas, VA, USA, Cat# 30-2002) supplemented with 10% FBS and 1% *v*/*v* penicillin/streptomycin. DTX-resistant PCa sublines were developed from low-passage cultures via continuous exposure to increasing concentrations of DTX (LC Laboratories, Woburn, MA, USA, Cat# D-1000), allowing for development of stable viability at each concentration until resistance was achieved to 10 nM DTX [54]. These sublines were maintained in medium containing 10 nM DTX, and resistance was monitored by 3-(4,5-dimethylthiazol-2-yl)-2,5-diphenyltetrazolium bromide MTT (Sigma-Aldrich, St. Louis, MO, USA, Cat# M5655) viability assay, and detection of multidrug resistance protein (MDR1/ABCB1) expression by immunoblotting. Cell lines were routinely tested for mycoplasma contamination using the MycoAlert Plus assay (Lonza, Walkersville, MD, USA, Cat# LT07-218), maintained at ≤80% confluency, and not allowed to exceed 28 passages. Identity of cell lines was authenticated by short tandem repeat analysis (STR; ATCC).

### 4.2. Detection of Nuclear GR and β-Catenin

Nuclear translocation of GR and β-catenin was assessed by treatment with dexamethasone (Sigma-Aldrich, St. Louis, MO, USA, Cat# D4902) followed by nuclear extraction using the CelLytic kit (Sigma-Aldrich, St. Louis, MO, USA, Cat# NXTRACT) and immunoblotting. Briefly, cells were seeded in 100 mm tissue culture dishes, allowed to adhere in humidified 37 °C/5% CO_2_ incubator for 24 h, and then incubated for 12 h in medium supplemented with 10% charcoal-stripped FBS (CS-FBS, Fisher Scientific, Pittsburgh, PA, USA, Cat# 12676-029) prior to treatment with dexamethasone. Cells were then trypsinized, washed with DPBS (ThermoFisher Scientific, Waltham, MA, USA, Cat# 14190144), and centrifuged at 300× *g* for 5 min at 4 °C. Pellets were resuspended in hypotonic lysis buffer supplemented with protease inhibitor cocktail (PIC, 1/100) (Sigma-Aldrich, St. Louis, MO, USA, Cat# NXTRACT) and incubated on ice for 15 min. IGEPAL^®^CA-630 (0.6%) (Sigma-Aldrich St. Louis, MO, USA, Cat# I8896) was added, and tubes were vortexed for 10 s and centrifuged at 11,000× *g* for 30 s at 4 °C. Supernatants containing cytoplasmic fraction were transferred to pre-chilled tubes. Pellets containing nuclei were resuspended in PIC-supplemented extraction buffer, vortexed for 20 min, and centrifuged at 20,000× *g* for 5 min at 4 °C. Supernatants containing soluble nuclear proteins were transferred to pre-chilled tubes prior to SDS-PAGE and immunoblotting.

### 4.3. Validation of Antibody Specificities via siRNA-Mediated Knockdown

Since GR and β-catenin have similar electrophoretic migration (94 and 92 kDa, respectively), we performed small inhibitory RNA (siRNA)-mediated knockdown of GR or β-catenin to rule out cross-reactivity of commercial antibodies used for co-immunoprecipitation (co-IP) and to explore whether the expression of these two proteins is inter-dependent. Non-specific scrambled oligo (Scr, 100 nM) or 100 nM total tri-silencer siRNA against GR (Origene Technologies, Rockville, MD, USA, Cat# SR301960) or 100 nM siRNA against β-catenin. SiRNA sequences specific for GR are (A) 5′-AGAAUGACCUACAUCAAAGAGCUAG, (B) 5′- GGAUACUAUACAAG CAGAACUGAGG, and (C) 5′-GGAGAUCAUAUAGACAA UCAAGUGC. Sequences specific for β-catenin (siRNA-1512) are sense 5′-GGGUUCC GAUGAUAUAAAUTT, antisense 5′-AUUUAUAUCAUCGGAACCCTT [83]. Briefly, cells were seeded in 6-well tissue culture plates and allowed to adhere in humidified 37 °C/5% CO_2_ incubator for 24 h. Cells were then washed with DPBS, resuspended in RPMI 1640 supplemented with 10% FBS in the absence of antibiotics, and returned to the incubator for 1 h. Transfection complexes were prepared using non-supplemented RPMI 1640, Oligofectamine (ThermoFisher Scientific, Waltham, MA, USA, Cat# 12252011), and 100 nM Scr control oligo, 33.3 nM each tri-silencer oligos A, B, and C specific for GR, or 100 nM siRNA against β-catenin. Complexes were incubated for 20 min at room temperature and added dropwise to cells. Plates were incubated for 48 h at 37 °C/5%CO_2_ followed by preparation of whole cell lysates by washing cells with ice-cold DPBS and scraping them on ice in Laemmli lysis buffer supplemented with complete PIC and 100 mM phenylmethanesulfonyl fluoride (PMSF)(Sigma-Aldrich, St. Louis, MO, USA, Cat# 329-98-06). Lysates were incubated on ice for 30 min, vortexing periodically, then sonicated and individually passed through a 22-gauge Hamilton syringe (Hamilton Company, Reno, NV, USA, Cat# 80565) until viscosity was minimal. Samples were centrifuged at 12,000 rpm for 5 min at 4 °C, and soluble proteins were used for SDS-PAGE and immunoblotting.

### 4.4. In Silico Analysis of GR and β-Catenin Protein Interaction Networks

This bioinformatics analysis was conducted using the STRING platform for protein–protein interaction networks and functional enrichment analysis (https://string-db.org/cgi/about (accessed on 14 March 2023)). The search focused on GR, using the gene name *NR3C1*, and β-catenin, using *CTNNB1*.

### 4.5. Co-Immunoprecipitation of GR and β-Catenin

Co-IP of GR and β-catenin was performed from whole cell lysates using an immunoprecipitation kit (Abcam, Waltham, MA, USA, Cat# ab206996). Briefly, cells were seeded in 100 mm tissue culture dishes, incubated for 24 h, washed with ice-cold DPBS, scraped in non-denaturing lysis buffer containing 1/500 PIC on ice, and collected into pre-chilled 1.5 mL microcentrifuge tubes. Tubes were set on a rotary mixer for 30 min at 4 °C and subsequently centrifuged at 10,000× *g* for 10 min at 4 °C. 500 μg of supernatant protein was incubated for 12 h on the rotary mixer at 4 °C with pre-washed Protein A/G Sepharose beads in 50% slurry in DPBS and 1:100 diluted antibody. Antibodies used for co-IP included normal rabbit IgG (EMD Millipore, Burlington, MA, USA, Cat# 3059594) as negative control, rabbit monoclonal anti-GR (Cell Signaling Technology, Danvers, MA, USA, Cat# 12041S, clone D6H2L), or rabbit monoclonal anti-total β-catenin (Cell Signaling Technology, Danvers, MA, USA, Cat# 8480S, clone D10A8). Antigen/antibody/bead samples were centrifuged at 2000× *g* for 2 min at 4 °C, and bead-bound complexes were washed three times with DPBS. Proteins were eluted by adding 4x lithium dodecyl sulfate (LDS) buffer (ThermoFisher Scientific, Waltham, MA, USA, Cat# NP0007) containing 1% β-mercaptoethanol and boiling for 5 min. Samples were centrifuged at 12,000 rpm for 3 min at 4 °C prior to SDS-PAGE.

Co-IP of nuclear GR and β-catenin was performed using the Nuclear Complex Co-IP kit (Active Motif, Carlsbad, CA, USA, Cat# 54001). Briefly, cells were seeded in 100 mm tissue culture dishes and allowed to adhere in a humidified 37 °C/5% CO_2_ incubator for 24 h. Cells were washed once with ice-cold PBS supplemented with freshly prepared 1/20 phosphatase inhibitor cocktail, then scraped gently into fresh, ice-cold PBS + phosphatase inhibitors, transferred to pre-chilled tube, and centrifuged for 5 min at 430× *g* at 4 °C. The supernatant was discarded, and the pellet was resuspended in hypotonic lysis buffer and incubated for 15 min on ice. Following addition of kit detergent, samples were mixed by gentle pipetting and centrifuged for 30 s at 14,000× *g* at 4 °C. The nuclear pellet was resuspended by gentle pipetting in the kit’s Complete Digestion Buffer supplemented with 100 mM PMSF and 1/100 PIC, followed by addition of Enzymatic Shearing Cocktail. Samples were vortexed gently for 2 s followed by 90 min incubation at 4 °C. After this step, which releases soluble protein complexes from chromatin, 0.5 M EDTA (Active Motif, Carlsbad, CA, USA, Cat# 54001) was added to stop the reaction, and samples were vortexed gently for 2 s and placed on ice for 5 min. Samples were centrifuged for 10 min at 14,000× *g* at 4 °C. Next, 100 μg of sample was combined with antibody diluted 1/100 in 500 μL of IP Incubation Buffer supplemented with PIC, and the antibody/extract mixture was incubated for 12 h in a rotator at 4 °C. Pre-washed Protein A/G Sepharose beads (Abcam, Waltham, MA, USA, Cat# 206996) were then added to the antibody/extract mixtures, which were again rotated for 1 h at 4 °C. Samples were then centrifuged at 1430× *g* for 30 s at 4 °C and the supernatant was discarded. Antibody/extract/bead complexes were washed three times with ice-cold IP Wash Buffer supplemented with 1 mg/mL bovine serum albumin (BSA), followed by three washes with ice-cold IP Wash Buffer without BSA. Pellets were resuspended in 4x LDS buffer, boiled for 5 min, and centrifuged at 12,000 rpm for 3 min at 4 °C prior to SDS-PAGE.

### 4.6. Electrophoresis and Immunoblotting

Cell lysates boiled in LDS sample buffer with reducing agent (Fisher Scientific, Pittsburgh, PA, USA, Cat# NP0004) were loaded into individual lanes (20 μg/lane) of 4–12% bis-tris SDS-polyacrylamide gels and electrophoresed in 1X MOPS running buffer (Fisher Scientific, Pittsburgh, PA, USA, Cat# NP0001) containing antioxidant (Fisher Scientific, Pittsburgh, PA, USA, Cat# NP0005) at constant 175 volts for 70 min. Proteins were then transferred to Immobilon-FL polyvinylidene fluoride (PVDF) membranes (EMD Millipore, Burlington, MA, USA, Cat# IPFL00010) in transfer buffer (Fisher Scientific, Pittsburgh, PA, USA, Cat# NP00061) for 90 min at constant 25 volts. Protein transfer was assessed by Ponceau S staining. Membranes were blocked for 1 h in tris-buffered saline containing 0.05% tween-20 (TBS-T) and 5% dry milk followed by incubation with primary antibodies for 12 h at 4 °C. Primary antibodies included mouse monoclonal anti-GR (BD Biosciences, Franklin Lakes, NJ, USA, Cat# 611226,) and anti-total β-catenin (BD Biosciences, Franklin Lakes, NJ, USA, Cat# 610153); rabbit monoclonal anti-non phosphorylated (active) β-catenin (Cell Signaling Technology, Danvers, MA, USA, Cat# 8814S, clone D13A1), anti-AR (Cell Signaling Technology, Danvers, MA, USA, Cat# 5153S, clone D6F11) and anti-AR-V7 (Cell Signaling Technology, Danvers, MA, USA, Cat# 19672S, clone E3O8L); rabbit polyclonal anti-histone H3 (GeneTex, Irvine, CA, USA, Cat# GTX122148), anti-MDR1/ABCB1 (Cell Signaling Technology, Danvers, MA, USA, Cat# 13342S, clone E1Y7B), and anti-Lens Epithelium Derived Growth Factor p75 (LEDGF/p75, Bethyl Laboratories/Fortis Life Sciences, Montgomery, TX, USA, Cat# A300-848A); rabbit monoclonal anti-GAPDH (Cell Signaling Technology, Danvers, MA, USA, Cat# 2118S, clone 14C10) and anti-alpha/beta-tubulin (Cell Signaling Technology, Danvers, MA, USA, Cat# 2148S), or horseradish peroxidase (HRP)-conjugated rabbit monoclonal anti-β-actin (Cell Signaling Technology, Danvers, MA, USA, Cat# 5125S clone 13E5). After incubation with primary antibodies, membranes were washed with TBS-T, followed by incubation for 1 h with appropriately diluted HRP-linked secondary antibodies, anti-rabbit IgG (Cell Signaling Technology, Danvers, MA, USA, Cat# 7074S), or anti-mouse IgG (Cell Signaling Technology, Danvers, MA, USA, Cat# 7076S). Membranes were washed with TBS-T, and immunoreactive bands detected with SuperSignal West Pico PLUS Chemiluminescent reagents in autoradiography film (Midwest Scientific, Fenton, MO, USA, Cat# XC6A2). Signals were quantified using ImageJ (https://imagej.nih.gov/ij/ (accessed on 23 March 2023)).

### 4.7. MTT Assays

Cells were seeded in triplicate in flat-bottom, tissue culture-treated 96-well plates and allowed to adhere in humidified 37 °C/5% CO_2_ incubator for 24 h. Subsequently, cells were treated with 10^−10^, 10^−9^, 10^−8^, 10^−7^, 10^−6^, or 10^−5^ M DTX, followed by incubation for 72 h to determine IC_50_ in sensitive and chemoresistant PCa cell lines. To investigate the effect of single and dual inhibition of GR and β-catenin on short-term cell viability, both DTX-sensitive and -resistant PCa cells were seeded in 96-well plates in normal growth medium and allowed to attach for 24 h. The normal medium was then changed to CS-FBS supplemented medium containing 10 nM dexamethasone. Cells were then treated with 1 μM of the β-catenin inhibitor methyl 3-([4-methylphenyl]sulfonyl]amino) benzoate MSAB (Sigma-Aldrich, St. Louis, MO, USA, Cat# SML-1726), 1 μM of the selective GR modulator CORT-108297 (AChemBlock, Hayward, CA, USA, Cat# L13976), or both inhibitors combined (1 μM each). Chemoresistant PCa cells were treated with inhibitors in the presence of 10 nM DTX to evaluate if inhibition of GR and β-catenin resensitized cells to DTX-induced cytotoxicity. Dexamethasone (10 nM) was present throughout the duration of the treatments. Following treatment, 25 μL of 5 mg/mL MTT solution in PBS was added per well, and plates were incubated for 2 h at 37 °C. Plates were centrifuged for 5 min at 1500 rpm, and supernatants removed and discarded. The MTT metabolite formazan within the cells was subsequently solubilized with DMSO (100 μL per well) on a plate shaker for 10 min at room temperature. Absorbance at 490 nm was measured using a SpectraMax spectrophotometer (Molecular Devices LLC, San Jose, CA, USA). Readings were normalized to vehicle-treated values.

### 4.8. Pharmacological Targeting of GR and β-Catenin in Adherent and Tumorsphere Cultures

Adherent PCa cells were treated with CORT-108297, MSAB, or both, followed by viability assessment as described above. Cells were also seeded in 6-well tissue culture plates, allowed to adhere for 24 h, washed, and changed to CS-FBS-supplemented medium containing vehicle (DMSO), CORT-108297 (1 μM), MSAB (1 μM), or CORT-108297 + MSAB (1 μM each). DTX-resistant cell cultures contained 10 nM DTX. All treatments were done in the presence of 10 nM dexamethasone. Cells were incubated for 72 h and visualized in an Olympus IX70 microscope equipped with Hoffman Modulation Contrast and SPOT imaging system.

To determine the effects of targeting GR and β-catenin in DTX-resistant PCa tumorspheres, we grew 3D cultures using Tumorsphere XF Cancer Stem Cell medium (PromoCell GmbH, Heidelberg, Germany, Cat# C-28070). Briefly, adherent DTX-resistant PCa cells were harvested at 75% confluency and seeded at 10,000 cells/mL in Tumorsphere XF medium containing either vehicle (DMSO), CORT-108297 (1 μM), MSAB (1 μM), CORT-108297 plus MSAB (1 μM each), or the BET bromodomain inhibitor JQ1 (1 μM) (MedChemExpress, Monmouth Junction, NJ, USA, Cat# HY-13030), and plated onto 60 mm ultra-low attachment dishes (Corning, Glendale, AZ, USA, Cat# 3261). Dishes were incubated in a humidified 37 °C/5% CO_2_ incubator, and tumorspheres were grown for 6 days. Tumorspheres were imaged as indicated above and then collected by gravity-facilitated sedimentation, trypsinized (PromoCell GmbH, Heidelberg, Germany, Cat# C-41010) for 90 s followed by neutralization with Trypsin Neutralizing Solution (PromoCell GmbH, Heidelberg, Germany, Cat# C-41110). Cells were then washed with PBS and stained for CSC-associated markers using mouse anti-human CD44 (BD Biosciences, Franklin Lakes, NJ, USA, Cat# 561292, clone G44-26) or anti-human CD24 (BD Biosciences, Franklin Lakes, NJ, USA, Cat# 555428, clone ML5) prior to flow cytometry [25].

### 4.9. Statistical Analysis

Two-sample comparisons were performed using Student’s *t*-tests, and multiple comparisons were performed using one-way ANOVA with GraphPad Prism, version 6.0c. Statistical significance was determined at *p* < 0.05.

## 5. Conclusions

This study provides evidence for GR and β-catenin nuclear accumulation in DTX-resistant cells in response to dexamethasone, nuclear GR/β-catenin interaction in mCRPC cells, and re-sensitization of DTX-resistant PCa cells and tumorspheres to DTX by pharmacological co-inhibition of these two proteins. While these results suggest that the GR/β-catenin interaction influences PCa stemness and chemoresistance, future studies would be necessary to demonstrate unambiguously that these features of aggressive prostate tumors are driven by this interaction andnot by the independent functions of the GR and Wnt/β-catenin pathways. These studies would provide a strong rationale for exploring the efficacy of blocking this interaction for depleting CSC populations and abrogating therapy cross-resistance in mCRPC.

## Data Availability

The data presented here are available on request from the corresponding author.

## Supplementary Materials

The supporting information can be downloaded at: https://www.mdpi.com/article/10.3390/ijms24087130/s1.
