# Supplementary material for: Glucocorticoid Receptor and β-Catenin Interact in Prostate Cancer Cells and Their Co-Inhibition Attenuates Tumorsphere Formation, Stemness, and Docetaxel Resistance
Source: Int J Mol Sci. 2023 Apr 12;24(8):7130. doi: 10.3390/ijms24087130 (PMC10139020; doi:10.3390/ijms24087130)
Supplement: Supplementary file 1 [file ijms-24-07130-s001.zip › ijms-2274896-supplementary.pdf]

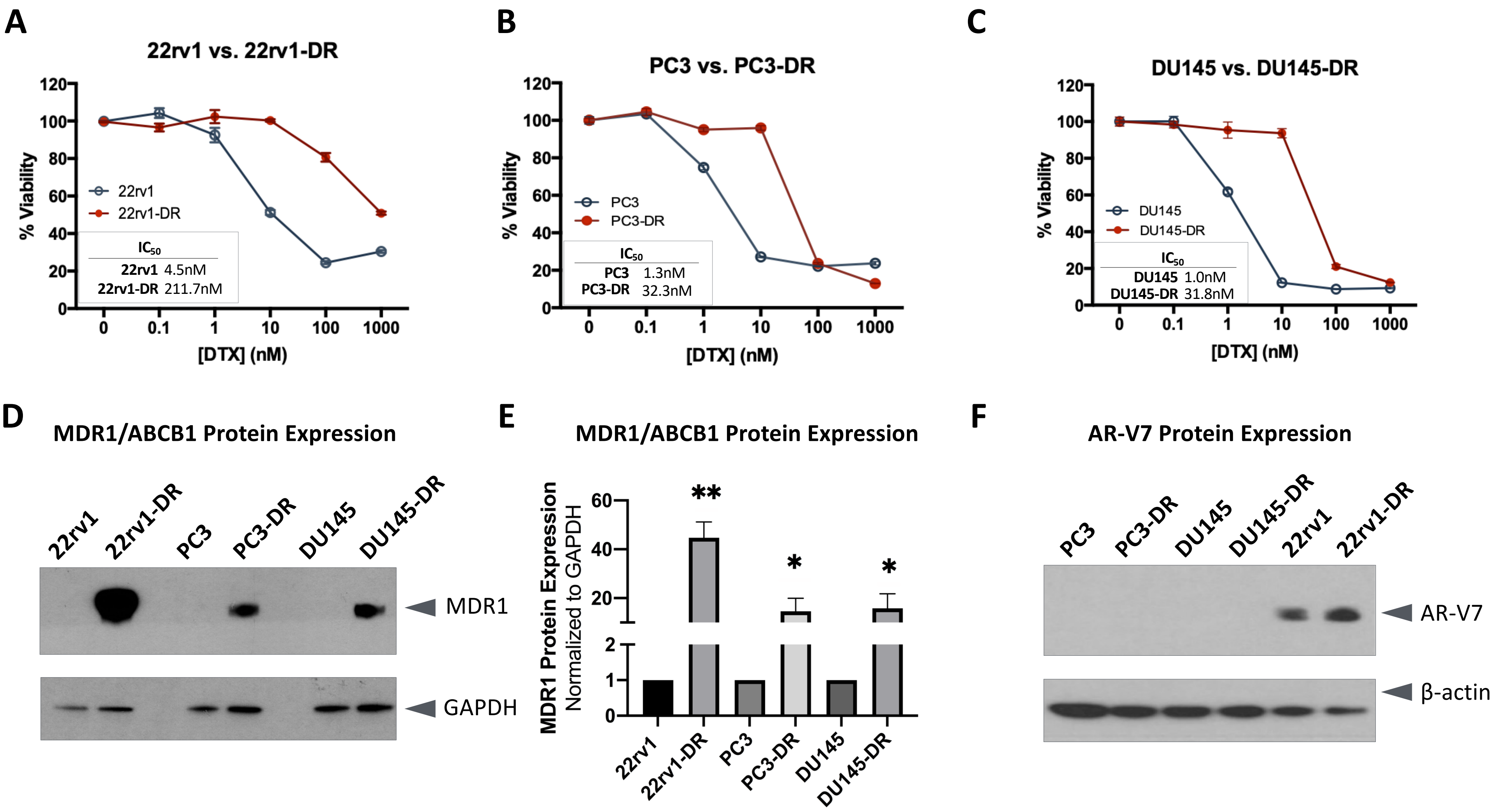

Figure S1. Characterization of docetaxel-resistant (DR) cell lines. **(A-C)** Validation of resistance to 10nM DTX in PC3-DR, DU145-DR, and 22rv1-DR cells by MTT assay following treatment for 72 hours. DR cells were generated by continuous exposure to incrementally increasing concentrations of DTX (0.1, 1, 3, 5, 6, 7, 8, 9, 10 nM) until stable (>95%) viability was achieved at 10 nM DTX. Data include at least 4 independent experiments and are represented as mean ± SEM. **(D,E)** Immunoblot and quantification of multidrug resistance protein (MDR1), also known as ATP binding cassette subfamily B1 (ABCB1) or p-glycoprotein, in DTX-sensitive and DTX-resistant cell lines. **(F)** Immunoblot showing increased AR-V7 variant in DTX-resistant 22rv1-DR cells compared to sensitive parental cells. Data include at least 3 independent sample sets and are represented as mean ± SEM. * $p < 0.05$, ** $p < 0.01$

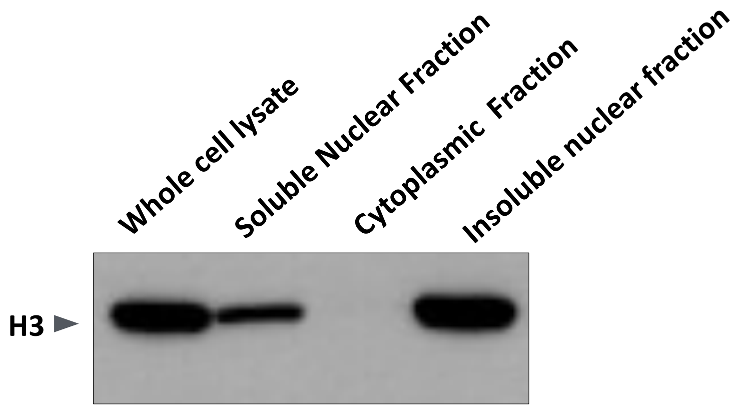

Figure S2. Validation of subcellular fractionation. Cellular fractionation studies were conducted as described in Materials and Methods. Histone H3 was detected in the soluble and insoluble nuclear fractions but not in the cytoplasmic fractions.

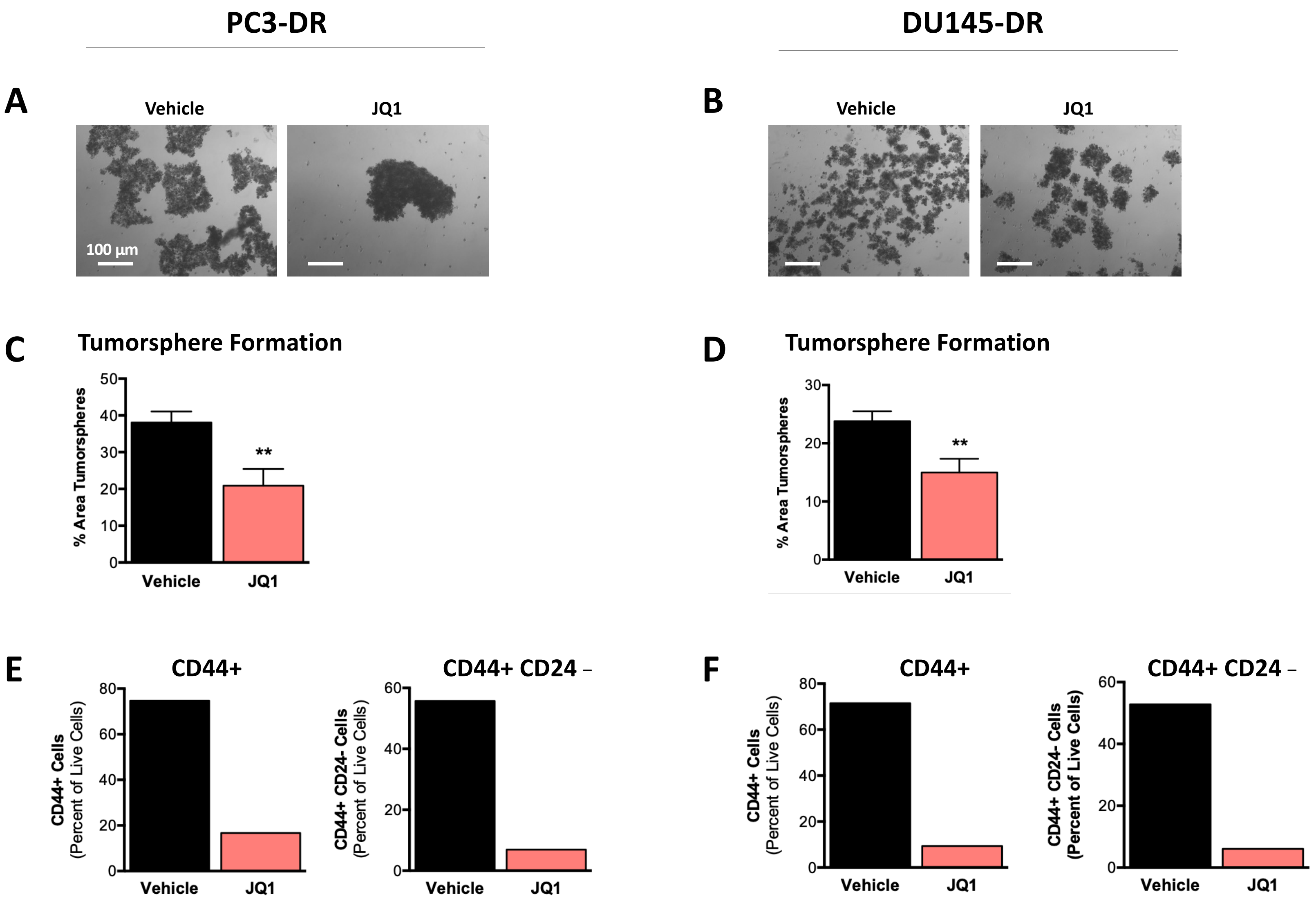

Figure S3. The BRD4 inhibitor JQ1 reduces tumorsphere formation and stemness in docetaxel-resistant cells. **(A,B)** PC3-DR and DU145-DR cells were cultured as tumorspheres in ultra-low adherency dishes in Tumorsphere XF medium containing 10 nM DTX and vehicle (DMSO) or the BRD4 inhibitor JQ1 (1 µM) for 6 days and imaged using an Olympus IX70 microscope. The scale bar was set at 100 µm for all the images. **(C,D)** Tumorsphere area was quantified from triplicate images per experiment using the ImageJ software. Data include at least 3 independent experiments and are represented as mean ± SEM (** $p < 0.01$). **(E,F)** CD44+ and CD44+ CD24– expression, presented as percent of live cells, from tumorspheres treated with JQ1 (1 µM) compared with vehicle control.
